# The potential of emerging bio-based products to reduce environmental impacts

**DOI:** 10.1038/s41467-023-43797-9

**Published:** 2023-12-21

**Authors:** Emma A. R. Zuiderveen, Koen J. J. Kuipers, Carla Caldeira, Steef V. Hanssen, Mitchell K. van der Hulst, Melinda M. J. de Jonge, Anestis Vlysidis, Rosalie van Zelm, Serenella Sala, Mark A. J. Huijbregts

**Affiliations:** 1 Department of Environmental Science, Radboud Institute for Biological and Environmental Sciences, Radboud University, Nijmegen, The Netherlands; 2 European Commission, Joint Research Centre, Ispra, VA Italy; 3 Department of Circularity & Sustainability Impacts, TNO, Utrecht, The Netherlands; 4 School of Chemical Engineering, National Technical University of Athens, Athens, Greece

**Keywords:** Environmental impact, Climate-change impacts, Climate-change mitigation

## Abstract

The current debate on the sustainability of bio-based products questions the environmental benefits of replacing fossil- by bio-resources. Here, we analyze the environmental trade-offs of 98 emerging bio-based materials compared to their fossil counterparts, reported in 130 studies. Although greenhouse gas life cycle emissions for emerging bio-based products are on average 45% lower (−52 to −37%; 95% confidence interval), we found a large variation between individual bio-based products with none of them reaching net-zero emissions. Grouped in product categories, reductions in greenhouse gas emissions ranged from 19% (−52 to 35%) for bioadhesives to 73% (−84 to −54%) for biorefinery products. In terms of other environmental impacts, we found evidence for an increase in eutrophication (369%; 163 to 737%), indicating that environmental trade-offs should not be overlooked. Our findings imply that the environmental sustainability of bio-based products should be evaluated on an individual product basis and that more radical product developments are required to reach climate-neutral targets.

## Abstract (web summary)

Zuiderveen and colleagues find that emerging bio-based products have on average 45% lower greenhouse gas life cycle emissions compared to their fossil counterparts, yet, there is a large variation between individual bio-based products with none of them reaching netzero emissions.

## Introduction

Many countries worldwide stimulate the development of a bio-based economy to mitigate climate change and to lower their dependency on fossil-based resources^1^. At the European level, the Bio-Economy Strategy^2^ was developed to guide Europe towards a sustainable bio-based economy, which was reinforced in the European Green Deal for achieving climate neutrality by 2050^3^. New bio-based products may improve environmental sustainability compared to their fossil counterparts^1^. A comprehensive meta-analysis on the environmental consequences of bio-based products compared to their fossil counterparts has, however, not been performed yet. More specific reviews are mainly in the domain of bio-plastics and question the claim of reduced environmental impacts^4–6^. Other reviews on biochemicals^7,8^ and bioadhesives^9,10^ show large variation between products for their climate change impacts and trade-offs regarding land use (change) and nutrient emissions.

Ensuring that bio-based products contribute to a sustainable economy requires comprehensive environmental assessments at an early stage of their development, considering the entire value chain, from feedstock sourcing and manufacturing, to the use phase and finally disposal. Prospective life cycle assessment (LCA) provides a method that can be applied to emerging products and technologies, i.e., with a technological readiness level (TRL) below 9, modelled to a future, more mature stage^11^. TRLs range from 1 to 9, from scientific breakthrough via lab development and pilot-phase, to a fully developed commercialized phase (TRL = 9)^12^. While an increasing number of prospective LCA studies has been carried out on emerging bio-based products^13–18^, their results vary strongly—not only due to differences in biomass feedstock and technology, but also due to the methodological challenges of prospective LCA^7^ and differences in biogenic carbon accounting^19,20^.

Here, we systematically compare the environmental footprints of 98 emerging bio-based products to their fossil-based counterparts reported in 130 prospective LCA studies. The analysis includes greenhouse gas (GHG) footprints as well as other environmental impacts (non-renewable energy-use, acidification, eutrophication, ozone depletion, and photochemical ozone formation). To allow intercomparison of environmental footprints, the system boundaries and biogenic carbon accounting are harmonized across studies. Environmental footprints are interpreted via response ratios (RR), which are defined as the natural-logarithm of the environmental impacts of the emerging bio-based product (*X*_*B*_) divided by its fossil counterpart (*X*_*F*_): RR = ln(X_B_/X_F_). The response ratios have a positive value (RR > 0) when the impact of the bio-based product is larger compared to its fossil-based counterpart, and a negative value (RR < 0) when the impact of the bio-based material is smaller. We determine average response ratios for each environmental impact using random-effects models to account for non-independence in data, i.e., accounting for multiple footprints from the same study and/or representing the same product. We also break down the analysis to evaluate systematic differences between (i) product category, (ii) feedstock category, and (iii) TRL. We quantify environmental trade-offs by studying differences in GHG, eutrophication, acidification, energy use, ozone depletion, and photochemical ozone formation footprints of bio-based products relative to their fossil-based counterparts. In the supplementary materials more details can be found on the main results and other environmental footprints.

## Results

### Greenhouse gas footprints

**Fig. 1:**
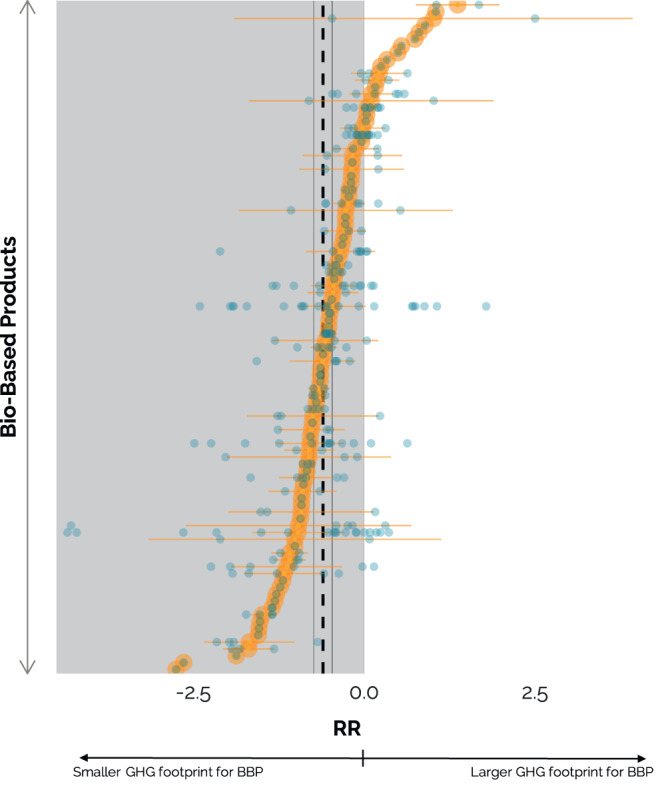
Scatterplot displaying all the response ratios as blue dots of the GHG footprints of bio-based products compared to their fossil counterparts, per bio-based product. Encircled orange dots represent arithmetic average RRs per bio-based product with corresponding 95% CI as opaque orange error-bars. There is no 95% CI for bio-based products with *n* = 1. Black dashed line at RR = −0.60 is the predicted mean RR based on a random-effects model including product type and study as random effects, accompanied by two black lines as overall 95% CI: −0.74 to −0.47. In the grey area, the GHG footprints of the bio-based products are lower than their fossil counterparts, with a grey line at RR = 0 representing no difference in GHG footprint. See Supplementary Table S. 3 for details.

The predicted mean of the bio-based products’ prospective GHG footprints are found to be 45% lower compared to their fossil-based counterparts (95% confidence interval (CI): −52 to −37%). Yet, the GHG footprints of emerging bio-based products vary widely compared to their fossil counterparts, ranging from a 294% (95% CI: 114% to 624%) higher footprint for lignin bioadhesives to a 94% (*n* = 1) lower footprint for wood fiber bio-composites compared to their fossil counterpart, as indicated in Fig. 1. Although, the majority of the bio-based products—80 in 98—show on average lower GHG footprints compared to their fossil counterparts, no product reaches net-zero GHG emissions. This suggests that most bio-based products thus reduce GHG emissions if they replace their fossil-based counterparts, but bio-based solutions are no guarantee for emission reduction and could in few cases in fact lead to higher GHG emissions.

When considering replacement of primary chemicals with bio-based alternatives in the chemical industry as a whole, significant GHG emission reduction may be achieved. The primary petrochemicals butadiene and ethylene are responsible for 34% of the primary chemical industry’s GHG emissions^21^. Replacing these with bio-based alternatives, which both have an arithmetic average reduction potential of 57% (95% CI: -71 to -37% for butadiene (*n* = 6), 95% CI: -73 to -32% for ethylene (*n* = 14)), could globally save up to 19% of the total GHG emissions from primary chemical production^22, 23^. The replacement of plastics, the most known petrochemical end product group, shows an average reduction potential of 38% (95% CI: -50 to -24%), which would result in saving 1.3% of the total global GHG emissions annualy^24^ (Supplementary Table S. 4). To achieve larger reductions of GHG emissions, increasing recycling rates, as well as electricity mixes dominated by renewable energy and electrification of the processes are crucial strategies that would not only benefit plastics^24,25^, but also all other types of products, both bio- and fossil-based.

### Product category

**Fig. 2:**
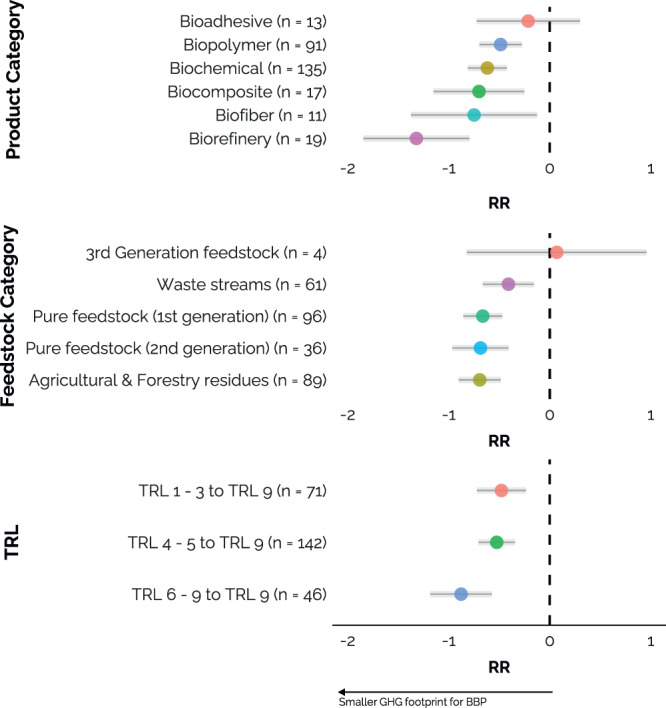
Change in GHG footprint response ratios (RR) in relation to key parameters. **a** Product category, **b** feedstock category and **b** TRL category, meaning the TRL from where the study up scales to a TRL 9. *n* gives the number of response ratios. Grey bars indicate 95% confidence intervals. Dashed black line at RR = 0 indicates no difference in GHG footprint between bio-based product and its fossil-based alternatives. In (**a**), biorefinery products refers to biochemicals produced in an integrated biorefinery producing multiple products and energy. For the results in (**c**), the 13 studies that did not model all the way up to a TRL 9 (but to a lower TRL, e.g. TRL 7) were excluded from the analysis. Plots show the predicted mean RR and 95% CI (error-bars) from single mixed-effects models. The predictions translated to percentages per category (in **a**–**c**) can be found in Supplementary Table S. 5.

The mean response ratio of biorefinery products, biochemicals, biocomposites, bioadhesives, and biopolymers are significantly different from zero, meaning the GHG footprints are lower compared to their fossil alternatives. Nevertheless, product category did not significantly explain variation in RRs (omnibus F: 2.13, *p*-value: 0.07). Still, the large reduction potential of biorefinery products is particularly promising, for which an average reduction of 73% (95% CI: –84 to –54%, Fig. 2a, *n* = 19) was found. Biorefineries produce multiple products in an integrated way, valorizing different parts of biomass feedstock and waste, and can therefore significantly lower the environmental footprint per product^26^.

The differences between the bio-product categories of biopolymers, biochemicals, biocomposites, biofibers and bioadhesives, were relatively small. Nonetheless, the category of bioadhesives (*n* = 13) stands out with the upper end of its confidence interval above zero. This result can be explained by the large influence of a single microalgae-based product that has a GHG footprint 12 times larger than its fossil counterpart due to high energy requirements of micro-algae cultivation and harvest^27^.

### Biomass feedstock category

The type of biomass feedstock used did not significantly influence the RRs and the differences between the feedstock categories were relatively small (except for 3^rd^ generation feedstock but *n* = 4), as shown in Fig. 2b (omnibus F: 1.53 *p*-value: 0.19). Although bio-based products from agricultural and forestry residues are on the lowest end, higher GHG emission reductions were expected for second generation feedstock compared to first generation biomass^28^. However, there is a wide variety in second generation biomass pretreatments, and some are significantly more intensive, e.g. in steam consumption or chemical use, compared to first generation biomass treatments^29^.

GHG emissions from land use change (LUC) can typically contribute a large share of the overall GHG footprint of bio-based products^30^ and biofuels^30–32^. Yet, in our analysis, only 13% of the studies included GHG emissions from LUC in their GHG footprint, and these did not necessarily result in systematically higher GHG footprints (Supplementary Fig. S. 6). The different methods used to arrive at LUC-related GHG emissions as well as the different types of original land that was transformed, makes it difficult to find a systematic effect. GHG emissions from LUC are highly variable, but can play a big role, specifically when deforestation is considered^33^. Future assessment of bio-based products should therefore include LUC-related GHG emissions, yet, currently, this is hindered by lack of a harmonized and standardized methodology^34^.

### Technology readiness level

Environmental footprints of emerging bio-based products are typically up scaled from the lab- or pilot-scale to a commercial stage. In the studies included in our review, the four main upscaling methods were via process simulation data (43% of the total number of data points), followed by adapted data from patents and reports (13%) or from similar processes that operate at large-scale (11%) and linear extrapolation of data (10%). We directly used the up scaled information in our analysis. We found that the starting TRL did not significantly influence the predicted GHG footprint of a technology, as the differences between the TRL groups were small and proved not significant (Fig. 2c; omnibus F: 2.26, *p*-value: 0.11). We should note, however, that predictions from lower TRLs that upscale to a TRL 9 should preferably involve a combination of process changes, size scaling and process synergies^35^. Although 91% of the studies up-scaled to commercial scale, based on production output or size, only 52% of them included one or more types of process synergies, such as heat integration, recovery of solvents, energy recovery from waste treatment or recycling of (waste) streams. Regardless of the original TRL, upscaling to a commercial stage can be more comprehensively assessed compared to the current state of the art.

### Environmental trade-offs

**Fig. 3:**
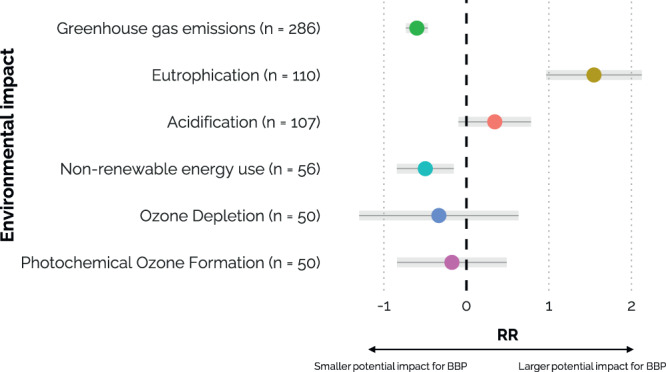
Plot showing predicted mean and 95% CI of GHG, eutrophication, acidification, NREU, ozone depletion and photochemical ozone formation impacts. In percentages, on average the GHG footprint is reduced by 45% (95% CI: −52 to −37%), eutrophication is increased with 369% (95% CI: 163 to 737%), acidification is increased with 41% (95% CI: −9 to 119%), NREU is reduced by 39% (95% CI: −57 to −14%), ozone depletion is reduced with 28% (95% CI: −73 to 88%) and photochemical ozone formation is reduced by 16% (95% CI: −57 to 63%). A plot with the arithmetic averages and 95% CIs can be found in Supplementary Fig. S. 9. A plot with an overview of all environmental impacts with n ≤ 30 and the predicted mean and 95% CI of the RRs across product types and studies can be found in Supplementary Fig. S. 10.

We found that emerging bio-based products have on average 37% lower (95% CI: -56 to -10%) non-renewable energy use (NREU) compared to their fossil counterparts, as shown in Fig. 3. In contrast, eutrophication impacts were on average 369% higher (95% CI: 163 to 737%) for bio-based products compared to their fossil counterparts. For the impact of acidification, ozone depletion and photochemical ozone formation, the bio-based products and their fossil alternatives were not significantly different from their fossil counterparts with a mean increase of 41% (95% CI: -9 to 119%), and mean reduction of 28% (95% CI: -73 to 88%) and 16% (95% CI: -57 to 63%) respectively.

Hence, for bio-based products to be more environmentally sustainable, total impacts should be minimized and burden shifting needs to be avoided, which mainly relates to the cultivation of biomass. Running linear mixed-effect models on the relationship between the RR of acidification and eutrophication impacts and feedstock categories, second generation feedstock did not seem to hold benefits over first generation feedstock (Supplementary Fig. S. 7). Second generation biomass may nevertheless hold benefits over first generation biomass in terms of food competition and biodiversity loss^36, 37^.

Exploring the relationship between the eutrophication and acidification impact and the product categories indicates a strong trade-off with climate change for all bio-based products. Only biorefinery products showed a decrease of 99% (95% CI: -100 to -94%) for acidification impacts compared to its fossil alternatives (Supplementary Fig. S. 6), though these results are relatively uncertain, due to the low number of biorefinery data points (*n* = 4). In general, eutrophication and acidification are highly influenced by the use of (synthetic) fertilizers^38^. Therefore, the use of more precise fertilization techniques, renewable fertilizers and sustainable agricultural practices are important to mitigate these impacts^39^.

## Discussion

To be able to combine the studies in a coherent statistical modeling format, we harmonized system boundaries, functional unit, end-of-life treatment and biogenic carbon accounting across all prospective LCAs. There are different ways of considering biogenic CO_2_, depending on the goal and scope of the study^40^. Most studies (83%) initially applied cradle-to-gate system boundaries, as chemical products are often intermediates applied in diverse downstream uses and therefore their end-of-life is unknown. For a consistent comparison across studies, we assumed that both biogenic and fossil carbon embodied in the products would eventually end up in the atmosphere, extending the system boundaries to cradle-to-grave by an incineration scenario based on the chemical structure of the product. We found that a large share of the products’ climate change impact relates to the embodied carbon in the product that is released again at the product’s end-of-life. Our end-of-life assumption that all products are incinerated may, however, overestimate the GHG emissions of both bio-based and fossil-based products that are currently recycled or biodegradable products that are converted into biogas, bio-energy or compost^4,7^. Moreover, the release of the embodied carbon can be abated by increasing the recycling rate^41,42^. We acknowledge that assessments of product design should ultimately integrate recycling, re-use or remanufacturing^43^. A closer collaboration between environmental and circularity assessments^44,45^ (e.g. the Material Circularity Indicator (MCI)^46^) which are yet to be standardized^44^, might be useful in this respect.

We found no indication that the starting TRL of a technology introduces systematic bias in the assessments. We were, however, not able to fully standardize the technology development predictions across the studies included, such as to what extent waste streams were recycled or heat integrated. To better understand the environmental impact of products at an early stage of development, clear upscaling guidelines involving different levels of technological development are required^47^. For instance, three distinctive steps in technological development can be specified, i.e. size scaling, process changes and process synergies^35^, which could then be assisted by for example expert views, simulation software and upscaling frameworks (e.g. from Piccinno et al.^48^). Additionally, a comprehensive prospective LCA would require temporal alignment of both the emerging technology and the fossil baseline product when compared at a future point in time, and should consider changes in the background system^47^. A standardized framework for prospective LCA might also benefit from clear TRL definitions. For this reason, there is first a need to develop per technology type specific TRL guidelines, which would make assessments much more comparable. Our study identified, for example, a broad application of fermentation-based technologies (55% of RRs), which will involve different (future) developments compared to thermochemical catalytic processes or integrated biorefineries.

The actual potential of bio-based products to reduce environmental impacts depends on scale and structure of the global bio-economy, which cannot be fully understood unless technology advancements are evaluated and, as crucial, the land-use change related emissions, which are typically not included in environmental assessment of bio-products, forming a crucial knowledge gap. Improved assessment of LUC related GHG emissions are also necessary to better understand the suggested advantages of second generation biomass (either dedicated crops or residues) over first generation biomass. Furthermore, only a limited number of studies included impacts on land use, water use and ecotoxicity from pesticide use. Yet, the studies reporting land- and water use indicate an increased impact for bio-based products (Supplementary Fig. S. 10). These categories also contribute to impacts on biodiversity. For example, agricultural cultivation can have a serious impact on biodiversity, e.g. converting natural habitat for palm oil cultivation leads to reductions in local wildlife populations and species richness^49^. Further research on all of these impacts is crucial to understand the sustainability of bio-based products.

Comparing prospective LCAs of emerging bio-based products to their fossil-based counterparts reveals a significant potential for the bio-economy to reduce GHG emissions. However, the large variability in GHG benefits and burdens of bio-based products compared to their fossil alternatives, with none of the products reaching net-zero emissions, asks for nuanced conclusions when designing and evaluating the sustainability of individual bio-based products. In the end, a combination of mitigation options like biomass utilization, increasing recycling rate and low carbon electrification of the industry, alongside reducing product demand^26,50,51^ will be required to reach net-zero emissions in the chemical and plastic industry.

## Methods

This section explains the data extraction process including search strategy, the screening and inclusion of prospective LCA studies and the framework developed to collect and harmonize data. The statistical analysis section describes the response ratio and the linear random- and mixed-effects models.

### Data extraction process

#### Search strategy

We searched for literature in Scopus and Web of Science (March 2023) using the search string: TI = ((lca) OR (life AND cycle AND assessment) OR (life AND cycle AND analysis) OR (environmental AND assessment) OR (environmental AND life AND cycle) OR (carbon AND footprint) OR (global AND warming AND potential) OR (cradle AND gate) OR (cradle-to-gate) OR (greenhouse gas) OR (GHG) OR (GWP)) AND TS = ((biochemical) OR (bio-chemical) OR (bioplastic) OR (bio-plastic) OR (biocomposite) OR (bio-composite) OR (biolubricant) OR (bio-lubricant) OR (biosurfactant) OR (bio-surfactant) OR (biopolymer) OR (bio-polymer) OR (biomaterial) OR (bio-material) OR (biofiber) OR (bio-fiber) OR (biobased) OR (bio-based) OR (bio AND based)). Additionally, a search string was used including: AND TS = (algae), to include studies using algae as a feedstock. There was no publishing year limit and we included all languages and document types. The search resulted in a total of 1349 studies, published between 1978 and 2023 (March 1^st^).

#### Screening and inclusion of prospective LCA studies

Based on abstract screening, we excluded studies on bio-based fuels to focus on emerging bio-based materials only, resulting in 428 studies. From these, 130 studies were selected for the analysis based on the following two criteria: (1) the study carried out an LCA with a prospective character, meaning the study assessed an emerging technology or material with a TRL below 9 modelled to a mature state in the future; and (2) the bio-based product is a ‘drop-in’ of a fossil-based product, meaning it has the same chemical structure, or it can be compared to a fossil-based product which has the same function (decision trees: Supplementary Fig. in S. 1).

#### Framework: collection and harmonization of prospective LCA results

To carry out the analysis, the studies were aligned concerning the biogenic carbon accounting and system boundaries. The following standardization approach was adopted:

1. Biogenic carbon emissions were considered CO_2_-neutral, because CO_2_ is taken up by growing biomass and released again at the end of the product life cycle. We consider this assumption defensible, as the considered biomass feedstock has a short rotation period—of typically one year (in line with the GWP_bio_ accounting approach by Cherubini et al.^51^) and temporary carbon storage in the bio-products is not considered relevant, as the materials considered are typically short-lived (in line with Guest et al.^20^), such as single use plastics.
2. The system boundary was set to cradle-to-grave by aligning end-of-life biogenic and fossil carbon emissions. From the papers, GHG emissions were extracted from cradle-to-gate (including biogenic carbon if it was subtracted from the GHG footprint at the gate) and an equal incineration end-of-life scenario based on the chemical structure of the product was added. Here, we accounted for CO_2_ emissions of the end-of-life incineration, but left all other waste treatment processes outside the system boundary, for both the bio-based products and their fossil-based counterparts.
3. For 10% of the studies the environmental impacts of the fossil-based counterparts were not given. These environmental impacts were calculated in SimaPro 9.1 software by applying impact assessment method matching the study’s impact assessment method (e.g. ReCiPe 2016^52^) on LCI datasets from Ecoinvent 3.7^53^ that fit within the same system boundaries.

For each study, we extracted the life cycle impact values of all the impact categories mentioned for the new bio-based products and its fossil-based alternatives^54^, either from the text, tables or graphs using WebPlotDigitizer (https://automeris.io/WebPlotDigitizer/). The categories of global warming, acidification, eutrophication, non-renewable energy use, ozone depletion and photochemical ozone formation contained a relatively large number of data values (*n* ≥ 50) and are displayed in Fig. 3. The predicted mean and 95% CI calculated for the other impact categories’ RRs (which all had *n* ≤ 30) can be found in Supplementary Fig. S. 10. The functional unit was taken as reported by the study with 95% of the studies applying functional units in weight (kg), 3% in area (m^2^) and 2% in volume (m^3^). We also included in our database (i) the product category (bioadhesive (incl. lubricants), biochemical, biocomposite, biofiber, biopolymer, biorefinery), (ii) the feedstock type (pure feedstock 1st generation, pure feedstock 2nd generation, agricultural & forestry residues, waste streams (including industrial side stream, municipal waste and food processing waste), 3rd generation), (iii) the original TRL (TRL 1 to 3, TRL 4 to 5, and TRL 6 to 9) and projected TRL. Definitions of the TRL groups were based on Moni et al.^12^ (Supplementary Fig. S. 2). For example, a study based on lab- or experimental data were considered a TRL 1 to 3, and a study based on process data by simulation of the design was considered a TRL 4 to 5.

### Statistical analysis

#### Response ratios

We calculated ln-response ratios to evaluate the change in environmental impacts between an emerging bio-based material and its fossil-based counterparts. The response ratio provides a measure of the relative change in environmental impacts. The response ratio (RR, dimensionless) was calculated as:

1 $${RR}={{{{\mathrm{ln}}}}}\left(\frac{{{{\mathrm{X}}}}_{{{\mathrm{B}}}}}{{\overline{{{\mathrm{X}}}}}_{{{\mathrm{F}}}}}\right)$$

where *x* is the environmental impact of the emerging bio-based product (B) and the fossil-based counterparts (F). Positive values for RR (RR > 0) indicate a larger footprint of the bio-based materials. Negative values for RR (RR < 0) indicate a smaller footprint of the bio-based materials. RR close to zero (RR ≈ 0), indicate no change in footprint. Throughout the text, the RR numbers are back-transformed using Euler’s number (e) and are reported as the percentage change from the fossil-based counterparts.

### Linear mixed models

Linear mixed models are an extension of regression models, and particularly useful for non-independency in data as they allow for random and fixed effects. If within one study the environmental impact of multiple products can be extracted, these footprints are non-independent. Hence, study identity is taken into account as random effect. Likewise, there are 98 different bio-based products. Footprints representing the same product are non-independent and therefore also taken into account as random effect. A random-effects model was ran to determine the mean RR across all studies and product types. Arithmetic average RRs were separately calculated per bio-based product with corresponding 95% confidence intervals (i.e., the ratio of the sum of RRs per bio-based product to the total number of corresponding bio-based product). Single linear mixed-effects models (LMM) were ran to assess the relationship between the RR of GHG, NREU, acidification, eutrophication, ozone depletion and photochemical ozone formation footprints and the key parameters, respectively product category, feedstock type and original TRL. Additional single linear mixed-effects model was ran to further explore the relationship between the RR of GHG footprints and GHG emission related to Land Use Changes (included/excluded in the study). Model fit of each of the mixed-effects models was assessed using the omnibus F test based on the Satterthwaite’s approximation to the denominator degrees of freedom. The analysis^54^ was carried out in *R v.4.1.3*^55^, using the *lme4* package^56^ to fit the LMM models, *lmerTest*^57^ to perform F-tests and *ggplot2*^58^ to generate figures.

### Supplementary information

Supplementary Information

## Data Availability

The supporting data generated in this study are provided in the Supplementary Information. The data collected in this study are available in the *figshare* repository (10.6084/m9.figshare.22795184)^54^.

## Code availability

The code to conduct the analysis is available in the *figshare* repository (10.6084/m9.figshare.22795184)^54^.
