# Supplementary material for: The potential of emerging bio-based products to reduce environmental impacts
Source: Nat Commun. 2023 Dec 21;14:8521. doi: 10.1038/s41467-023-43797-9 (PMC10739733; doi:10.1038/s41467-023-43797-9)
Supplement: Supplementary file 1 — Supplementary Information [file 41467_2023_43797_MOESM1_ESM.pdf]

**Supplementary**

# The potential of emerging bio-based products to reduce environmental impacts

Emma A. R. Zuiderveen[1,2*], Koen J. J. Kuipers[1], Carla Caldeira[2], Steef V. Hanssen[1], Mitchell K. van der Hulst[1,3], Melinda M.J. de Jonge[1], Anestis Vlysidis[2,4], Rosalie van Zelm[1], Serenella Sala[2], Mark A. J. Huijbregts[1,3]

[1] Department of Environmental Science, Radboud Institute for Biological and Environmental Sciences, Radboud University Nijmegen, P.O. Box 9010, 6500 GL Nijmegen, The Netherlands.

[2] European Commission, Joint Research Centre, Via Enrico Fermi 2749, 21027, Ispra, VA, Italy.

[3] Department of Circularity & Sustainability Impacts, TNO, Princetonlaan 6, 3584CB Utrecht, The Netherlands

[4] School of Chemical Engineering, National Technical University of Athens, Iroon Polytechneiou 9, Zografou, 15780 Athens, Greece

**Contents**

S.1 Methods: Decision Trees ........ 2

S.2 Methods: Overview TRLs ........ 4

S.3 Arithmetic average RRs per bio-based product with corresponding 95% CI: ordered from largest reduction to highest increase. ........ 5

S.4 Additional calculations in ‘Greenhouse gas Footprints’ ........ 7

S.5 Predicted mean reduction (RR) and 95% CI for GHG emissions per product category, feedstock category and TRL based on single linear mixed-effect models ........ 8

S.6 Predicted mean RR and 95% CI for the GHG emissions and if it in- or excluded Land Use Changes (LUC) related GHG emissions based on single linear mixed-effect models. ........ 8

S.7 Predicted mean RR and 95% CI per feedstock category based on single linear mixed-effect models for eutrophication, acidification, non-renewable energy-use, ozone depletion and photochemical ozone formation impacts. ........ 9

S.8 Predicted mean RR and 95% CI per product category based on single linear mixed-effect models for eutrophication, acidification, non-renewable energy-use, ozone depletion and photochemical ozone formation impacts. ........ 10

S.9 Overview of arithmetic average RRs and corresponding 95% CI for greenhouse gas emissions, eutrophication, acidification, non-renewable energy-use, ozone depletion and photochemical ozone formation impacts ........ 11

S.10 Overview of the predicted mean and 95% CI of the RRs across all product types and studies for all environmental impacts with n ≤ 30. Separate random-effects models were ran for each impact category. ........ 12

References ........ 13

Data References ........ 14

**Abbreviations**

| | |
|---|---|
| BBP | Bio-based products |
| GHG | Greenhouse gas |
| LCA | Life Cycle Assessment |
| LMM | Linear mixed-effects model |
| LUC | Land Use Change |
| NREU | Non Renewable Energy Use |
| RR | Response Ratio |
| TRL | Technology Readiness level |

| | |
|---|---|
| *Arithmetic average* | *The ratio of the sum to the total number* |
| *Predicted mean* | *The mean calculated by running LMM* |

## S.1 Methods: Decision Trees

The screening and inclusion of prospective LCAs followed two criteria. For the first criteria, we followed the decision tree in Figure S.1.1 on the scope, quality and clarity of the study. For the second criteria, we followed the decision tree in Figure S.1.2.

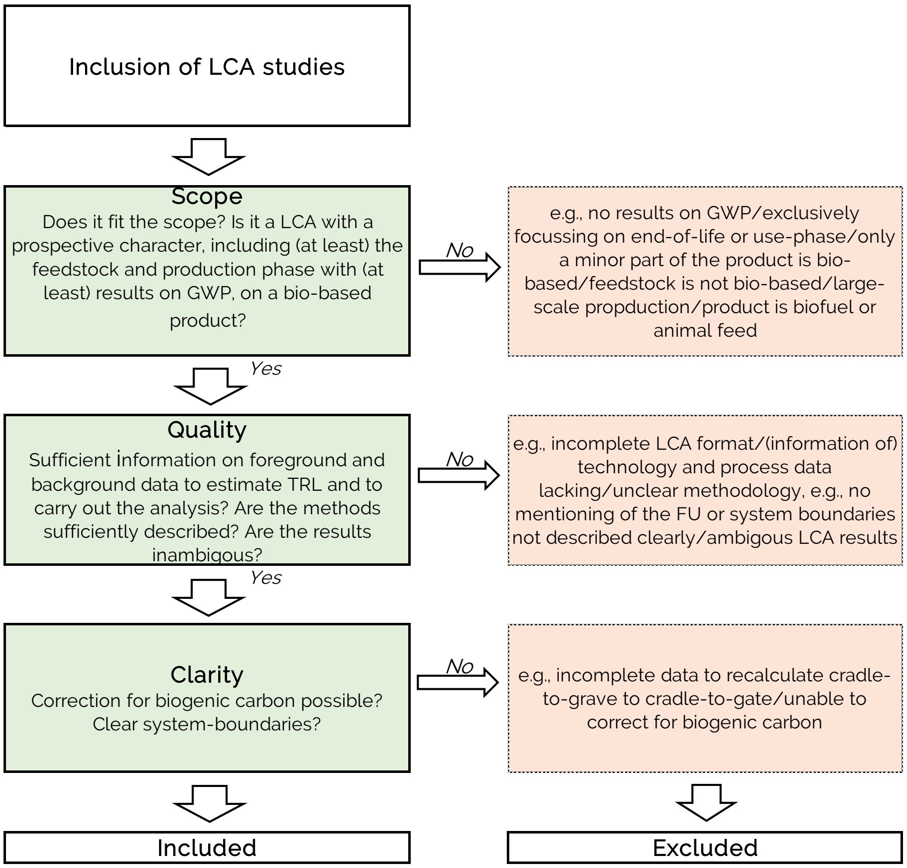


***Figure S.1.1**: Decision tree study selection related to the scope of the study. Furthermore, if the quality and/or clarity was lacking the study had to be excluded as well.*

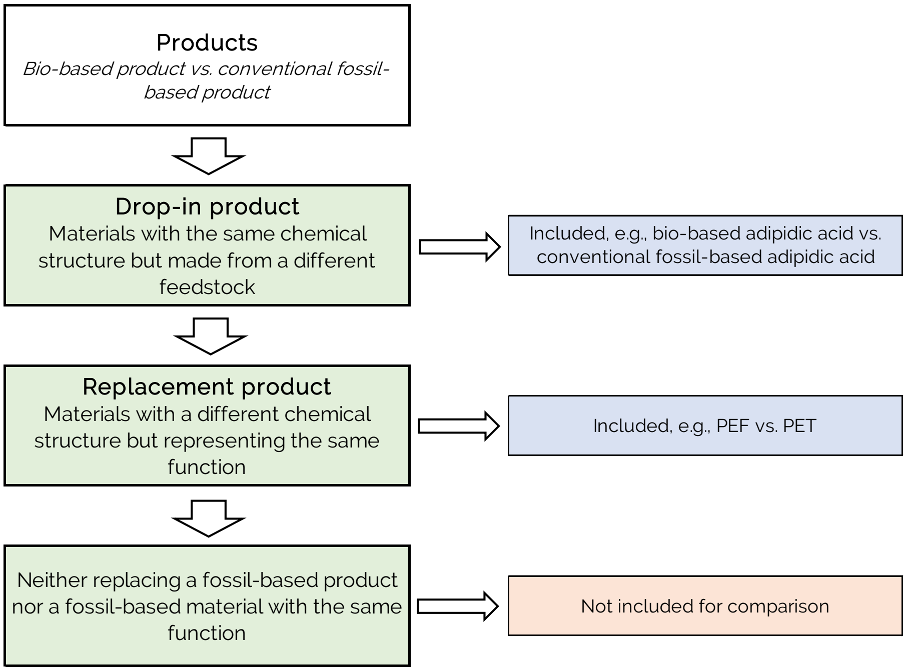


***Figure S.1.2**: Decision tree on products to include in the comparative assessment. The bio-based product is a 'drop-in' of a fossil-based product, meaning it has the same chemical structure, or it can be compared to a fossil-based product which has the same function*

## S.2 Methods: Overview TRLs

Only in a few cases the TRL was addressed by the study itself. The TRL evaluation was therefore based on the TRL specification from Moni et al. (2020)[1] and afterwards regrouped into broader category classes (TRL 1-3, TRL 4-5, TRL 6-9). Studies directly based on lab- or experimental data were assigned 'TRL 1-3'; studies based on simulations of the process, including downstream steps, were assigned 'TRL 4-5'; and studies in pilot phase or operating under expected conditions were assigned 'TRL 6-9'.

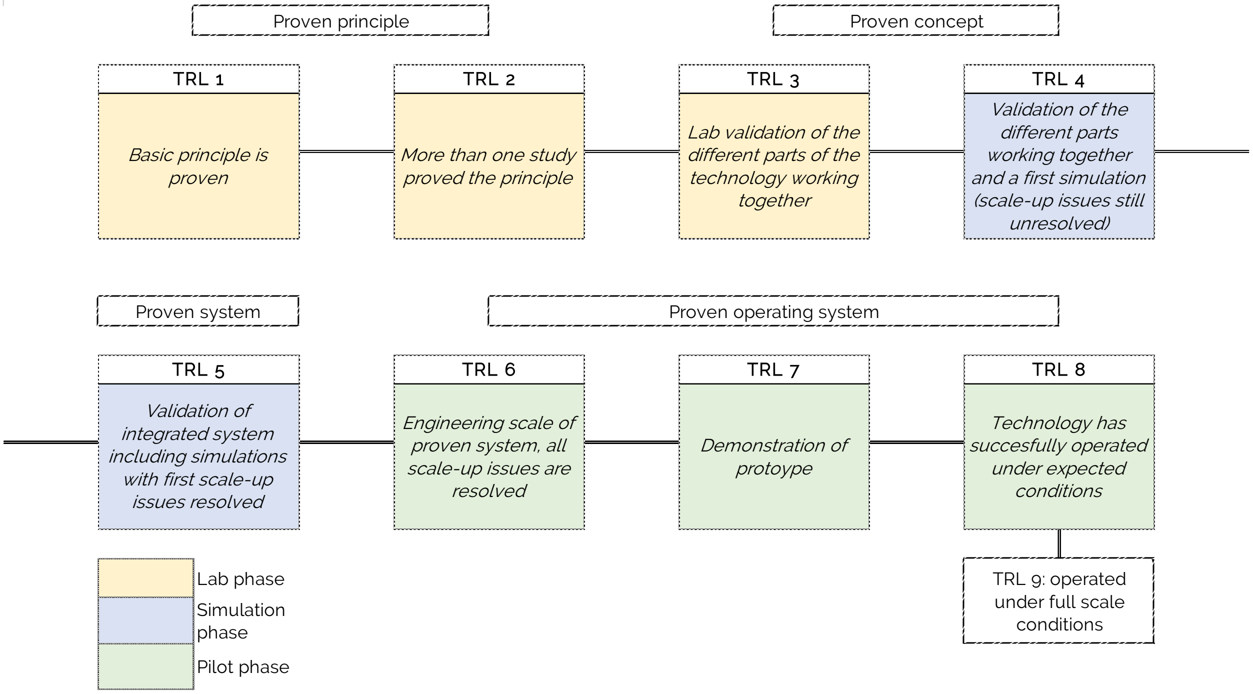


***Figure S.2**: Description of TRLs (Technology Readiness Level) as applied in this study*

## S.3 Arithmetic average RRs per bio-based product with corresponding 95% CI: ordered from largest reduction to highest increase.

***Table S.3**: overview of the bio-based products, the number of data points (n), the arithmetic average RR and 95% CI; if n = 1, there is no 95% CI, which is indicated with 0; *indicates the bio-based product with their average and 95% CI below zero.*

| Bio-based product | n | Arithmetic average (RR) | Lower end 95% CI | Higher end 95% CI |
|---|---|---|---|---|
| WOOD FIBRE/PLA/TPS BIOCOMPOSITE | 1 | -2.75 | 0.00 | 0.00 |
| WOOD FIBRE/PLA BIOCOMPOSITE | 1 | -2.64 | 0.00 | 0.00 |
| SPIRO-BISPHENOL | 1 | -1.87 | 0.00 | 0.00 |
| BOPLA | 3 | -1.69 | -2.06 | -1.32 |
| 1,3-PROPANEDIOL | 4 | -1.68 | -2.34 | -1.02 |
| 3-HYDROXY PROPIONIC ACID | 1 | -1.55 | 0.00 | 0.00 |
| BIOLUBRICANT | 1 | -1.54 | 0.00 | 0.00 |
| POLYLIMONENE CARBONATE | 1 | -1.52 | 0.00 | 0.00 |
| HEXANOIC ACID | 4 | -1.48 | -1.66 | -1.31 |
| BIOPOLYETHYLENE/BAGASSE FIBERS BIOCOMPOSITE | 2 | -1.35 | -1.36 | -1.33 |
| DIOL V | 1 | -1.30 | 0.00 | 0.00 |
| POLYLACTIDE | 1 | -1.28 | 0.00 | 0.00 |
| BIOPOLYMER | 1 | -1.23 | 0.00 | 0.00 |
| WAX ESTERS | 1 | -1.19 | 0.00 | 0.00 |
| ADIPIC ACID | 5 | -1.17 | -1.76 | -0.58 |
| NANOCELLULOSE (MICROFIBRILLATED CELLULOSE (MFC)) | 6 | -1.13 | -1.93 | -0.32 |
| POLYOLS | 3 | -1.09 | -1.33 | -0.85 |
| POLYESTER BINDERS | 2 | -1.09 | -1.36 | -0.81 |
| WHEAT-GLUTEN-BASED PACKAGING FILM | 1 | -1.01 | 0.00 | 0.00 |
| CELLULOSE NANOWHISKERS | 2 | -1.01 | -3.15 | 1.13 |
| LACTIC ACID | 18 | -0.97 | -1.64 | -0.29 |
| AROMATICS | 5 | -0.96 | -2.61 | 0.69 |
| FLAX MAT/PLA BIOCOMPOSITE LAMINATES | 1 | -0.93 | 0.00 | 0.00 |
| PEF | 3 | -0.92 | -1.99 | 0.14 |
| LINSEED OIL/FLAX FIBRE/MMP BIOCOMPOSITE | 1 | -0.92 | 0.00 | 0.00 |
| 3-PROPANEDIOL | 1 | -0.91 | 0.00 | 0.00 |
| POLYITACONIC ACID | 2 | -0.90 | -1.40 | -0.41 |
| SPIROCYCLIC DIOL | 1 | -0.89 | 0.00 | 0.00 |
| 1,3-BUTADIENE | 6 | -0.85 | -1.24 | -0.46 |
| LEVOGLUCOSAN | 1 | -0.84 | 0.00 | 0.00 |
| EPOXY RESIN SUPERSAP | 3 | -0.83 | -0.90 | -0.76 |
| PP | 3 | -0.80 | -2.01 | 0.40 |
| ISOBUTANOL | 2 | -0.80 | -1.17 | -0.44 |
| ETHYLENE | 13 | -0.79 | -1.28 | -0.30 |
| BIOETHANOL-BASED PVC | 1 | -0.79 | 0.00 | 0.00 |
| FORMIC ACID | 3 | -0.77 | -1.25 | -0.29 |
| CURAUÁ/PP COMPOSITE | 1 | -0.76 | 0.00 | 0.00 |
| PROPYLENE GLYCOL | 3 | -0.75 | -1.71 | 0.21 |
| 2,3-BUTANEDIOL | 4 | -0.70 | -0.81 | -0.60 |
| PE | 1 | -0.70 | 0.00 | 0.00 |
| MULCH FILM | 3 | -0.68 | -0.79 | -0.57 |
| 1,4-BUTANEDIOL | 2 | -0.66 | -0.82 | -0.50 |
| ALKYL POLYGLYCOSIDES | 1 | -0.64 | 0.00 | 0.00 |
| STARCH-FILLED POLYPROPYLENE | 2 | -0.64 | -0.65 | -0.63 |
| LIGNIN POWDER | 1 | -0.64 | 0.00 | 0.00 |
| PLA | 5 | -0.60 | -1.08 | -0.12 |

| | | | | |
|---|---|---|---|---|
| CURED WOOD FLOORING COATING | 1 | -0.60 | 0.00 | 0.00 |
| PROPIONIC ACID | 6 | -0.59 | -0.78 | -0.40 |
| TEREPHTHALIC ACID | 3 | -0.57 | -1.34 | 0.21 |
| LACTIDE | 8 | -0.53 | -0.56 | -0.50 |
| ACETONITRILE | 1 | -0.52 | 0.00 | 0.00 |
| OLEFINS | 1 | -0.52 | 0.00 | 0.00 |
| SODIUM POLYACRYLATE (NA-PA) | 2 | -0.50 | -0.52 | -0.48 |
| SUCCINIC ACID | 21 | -0.47 | -0.97 | 0.02 |
| PSA BIOADHESIVE | 1 | -0.47 | 0.00 | 0.00 |
| ETHYL LACTATE | 2 | -0.46 | -0.83 | -0.09 |
| PHA | 11 | -0.45 | -0.77 | -0.13 |
| POLYETHYLENE | 1 | -0.44 | 0.00 | 0.00 |
| N-VINYL-2-PYRROLIDONE | 4 | -0.41 | -0.50 | -0.32 |
| POLYURETHANE FOAM (PUF) | 4 | -0.41 | -0.54 | -0.28 |
| PU FOAMS | 1 | -0.37 | 0.00 | 0.00 |
| PHB | 8 | -0.34 | -0.85 | 0.16 |
| FDCA | 1 | -0.32 | 0.00 | 0.00 |
| PHB/KENAF COMPOSITE | 1 | -0.30 | 0.00 | 0.00 |
| CAPROIC ACID | 3 | -0.28 | -0.60 | 0.03 |
| ALGINATE-BASED PLASTIC | 1 | -0.27 | 0.00 | 0.00 |
| PHENOLIC | 1 | -0.27 | 0.00 | 0.00 |
| P-XYLENE | 2 | -0.27 | -1.84 | 1.30 |
| HDPE | 6 | -0.23 | -0.48 | 0.01 |
| PAG BIOADHESIVE | 1 | -0.22 | 0.00 | 0.00 |
| MODAL ANTI BACTERIAL FABRIC | 2 | -0.22 | -0.32 | -0.12 |
| BIOFILM | 1 | -0.19 | 0.00 | 0.00 |
| BUTYLCATECHOL | 1 | -0.19 | 0.00 | 0.00 |
| LLDPE | 2 | -0.18 | -0.95 | 0.58 |
| EPOXIDIZED SUCROSE SOYATE COMPOSITES | 1 | -0.17 | 0.00 | 0.00 |
| LDPE | 2 | -0.17 | -0.90 | 0.56 |
| 1,3-DIHYDROXYACETONE | 3 | -0.12 | -0.46 | 0.22 |
| SOY BIOADHESIVE | 1 | -0.04 | 0.00 | 0.00 |
| HEXAMETHYLENEDIAMINE | 12 | -0.03 | -0.10 | 0.05 |
| ISOBUTENE | 3 | -0.02 | -0.36 | 0.31 |
| TANNIN BIOADHESIVE | 1 | 0.03 | 0.00 | 0.00 |
| KETONE WAX | 1 | 0.04 | 0.00 | 0.00 |
| PET | 9 | 0.05 | -0.06 | 0.16 |
| METHANOL | 2 | 0.10 | -1.69 | 1.90 |
| PBS | 7 | 0.11 | -0.22 | 0.43 |
| PHLA | 1 | 0.16 | 0.00 | 0.00 |
| ACETONE | 2 | 0.19 | -0.14 | 0.52 |
| 2-METHYL TETRAHYDROFURAN | 3 | 0.22 | -0.19 | 0.63 |
| MANGO KERNEL STARCH FILM | 1 | 0.24 | 0.00 | 0.00 |
| POLY(LACTIC ACID)/CELLULOSE NANOCRYSTAL/LIMONENE | 1 | 0.34 | 0.00 | 0.00 |
| NANOFIBRILLATED CELLULOSE (NFC)-REINFORCED EPOXY | 1 | 0.49 | 0.00 | 0.00 |
| NANOCELLULOSE YARN | 1 | 0.55 | 0.00 | 0.00 |
| MYCOBAMBOO | 1 | 0.74 | 0.00 | 0.00 |
| BACTERIAL CELLULOSE (BC)-REINFORCED EPOXY COMPOSITES | 1 | 0.81 | 0.00 | 0.00 |
| SCG/PBS COMPOSITE | 1 | 0.89 | 0.00 | 0.00 |
| METAL WORKING FLUIDS (MWF) | 2 | 1.02 | -1.90 | 3.93 |
| CNC FOAM | 1 | 1.05 | 0.00 | 0.00 |
| LIGNIN BIOADHESIVE | 2 | 1.37 | 0.76 | 1.98 |

## S.4 Additional calculations in 'Greenhouse gas Footprints'

***Table S.4****: additional GHG emission calculations in the section 'Greenhouse gas Footprints'.*

| | **GHG emissions (Gt CO2 eq./yearly)** | **Percentage of global emissions yearly** | *Source* |
|---|---|---|---|
| **Total GHG emissions globally** | 52 | 100% | Ritchie, H. and M. Roser. $CO_2$ and Greenhouse Gas Emissions. *Our World in Data* (2020)[2] |
| **Total GHG emissions Plastics (fossil-based)** | 1.8 | 3.4% | Zheng, J. & Suh, S. Strategies to reduce the global carbon footprint of plastics (2019)[3] |
| **Total GHG emissions Primary Industry (based on the six main platform chemicals)** | 0.91 | 2% | Galán-Martín, Á. *et al.* Sustainability footprints of a renewable carbon transition for the petrochemical sector within planetary boundaries (2021)[4] |
| | **Ethylene** | **Butadiene** | **Plastics** |
| **Total amount produced yearly (kg)** | 2.01E11 | 1.2E10 | - |
| **GHG emissions fossil-based cradle-to-gate (kg CO2 eq. /kg chemical)** | 1.45 | 1.2 | - |
| **Bio-based potential reduction** | 57% (95% CI: 32, 73%) | 57% (95% CI: 37, 71%) | 38% (95% CI: -23, 50%) |
| **% of GHG emissions primary industry / global** | **18.3% / 0.3%** | **0.9% / 0.02%** | **- / 1.32%** |
| *Calculation* | $\frac{(RP_{BBP} \cdot GHG_{fossil}) \cdot T_{product}}{GHG_{total}} \cdot 100$ $with\ RP_{BBP}\ as\ biobased\ product\ reduction\ potential;\ GHG_{fossil}\ as\ GHG\ emissions\ fossilbased\ product;$ $T_{product}\ as\ total\ amount\ produced\ (kg/year);\ GHG_{total}\ as\ total\ GHG\ emissions\ (Primary\ Industry/Global)$ | | |
| *Sources* | Reduction potentials taken from S.1 and Figure 2a; Levi, P.G. and J.M. Cullen. 2018. Mapping Global Flows of Chemicals: From Fossil Fuel Feedstocks to Chemical Products[5]; Ecoinvent Centre. Ecoinvent database (Version 3.7)[6]. | | |

## S.5 Predicted mean reduction (RR) and 95% CI for GHG emissions per product category, feedstock category and TRL based on single linear mixed-effect models

***Table S.5****: Change in GHG footprint response ratios (RR) of bio-based products in comparison to fossil-based counterfactual in relation to key parameters: product category, feedstock category and TRL category (corresponding to Fig. 2 in the main text). Here, predictions with corresponding 95% confidence intervals are in percentages.*

| | n | Prediction [95% CI] |
|---|---|---|
| **Product category** | | |
| Bioadhesive | 13 | -19% [-52% to 35%] |
| Biochemical | 135 | -46% [-56% to -35%] |
| Biocomposite | 17 | -50% [-68% to -22%] |
| Biofiber | 11 | -53% [-75% to -12%] |
| Biopolymer | 91 | -38% [-50% to -24%] |
| Biorefinery | 19 | -73% [-84% to -55%] |
| | | |
| **Feedstock category** | | |
| 3rd generation feedstock | 4 | 7% [-56% to 161%] |
| Agricultural & forestry residues | 89 | -51% [-59% to -38%] |
| Waste streams | 61 | -33% [-48% to -14%] |
| Pure feedstock (1st generation) | 96 | -49% [-58% to -38%] |
| Pure feedstock (2nd generation) | 36 | -50% [-62% to -34%] |
| | | |
| **TRL** | | |
| TRL 1-3 | 71 | -38% [-51% to -21%] |
| TRL 4-5 | 142 | -41% [-51% to -29%] |
| TRL 6-9 | 46 | -58% [-69% to -44%] |

## S.6 Predicted mean RR and 95% CI for the GHG emissions and if it in- or excluded Land Use Changes (LUC) related GHG emissions based on single linear mixed-effect models.

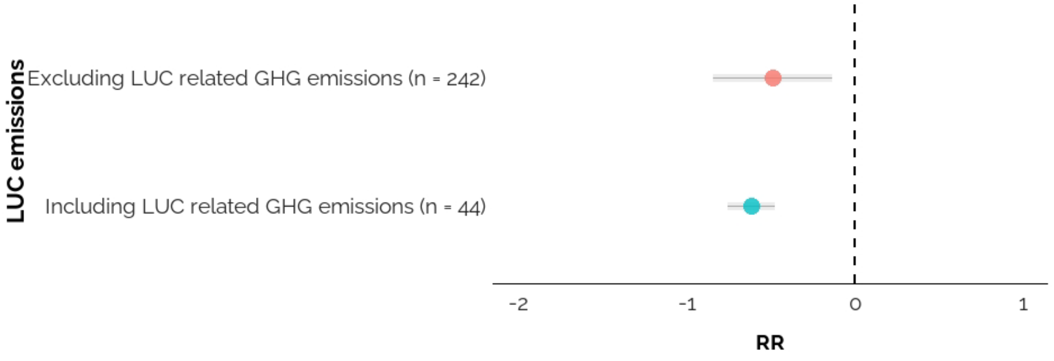


***Figure S.6****: LMM on relationship RR GHG emissions and studies either in- or excluding LUC. Average estimate including LUC is -46% (95% CI: 38, 53%) and excluding LUC is -39% (95% CI: 13, 57%). Omnibus F:0.44, p-value: 0.51.*

**S.7 Predicted mean RR and 95% CI per feedstock category based on single linear mixed-effect models for eutrophication, acidification, non-renewable energy-use, ozone depletion and photochemical ozone formation impacts.**

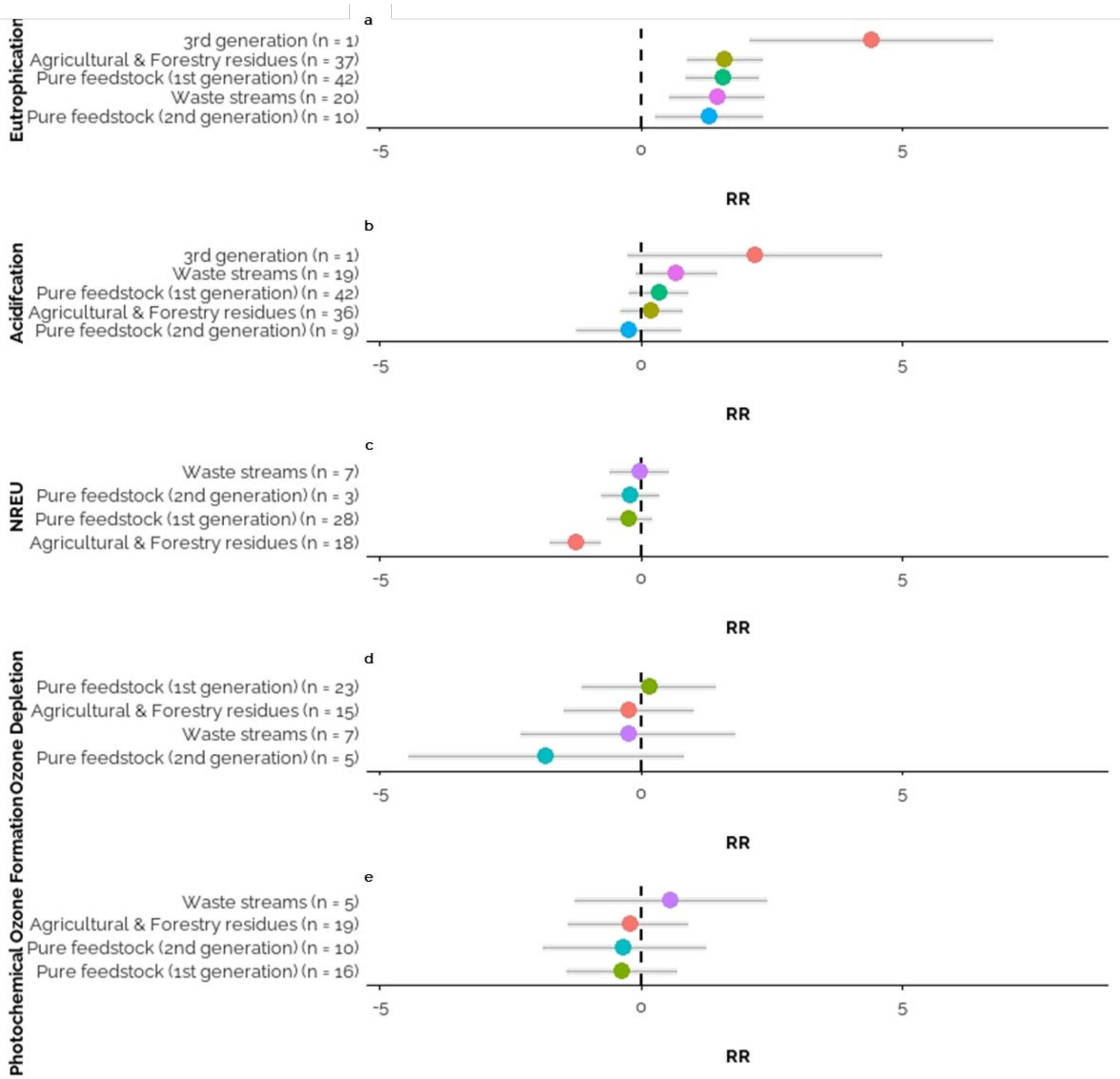


***Figure S.7**: LMM on relationship RR environmental footprints and feedstock category: (a) Eutrophication: omnibus F: 1.58, p-value: 0.19; (b) Acidification: omnibus F: 1.08, p-value: 0.37; (c) NREU: omnibus F: 7.32, p-value: 0.0006*; (d) Ozone Depletion: omnibus F: 0.65, p-value: 0.59; (e) Photochemical Ozone Formation: omnibus F: 0.25, p-value: 0.85. *For NREU, the feedstock category as single effect proved significant, but due to the low number of data points for each category we consider this result highly uncertain.*

**S.8 Predicted mean RR and 95% CI per product category based on single linear mixed-effect models for eutrophication, acidification, non-renewable energy-use, ozone depletion and photochemical ozone formation impacts.**

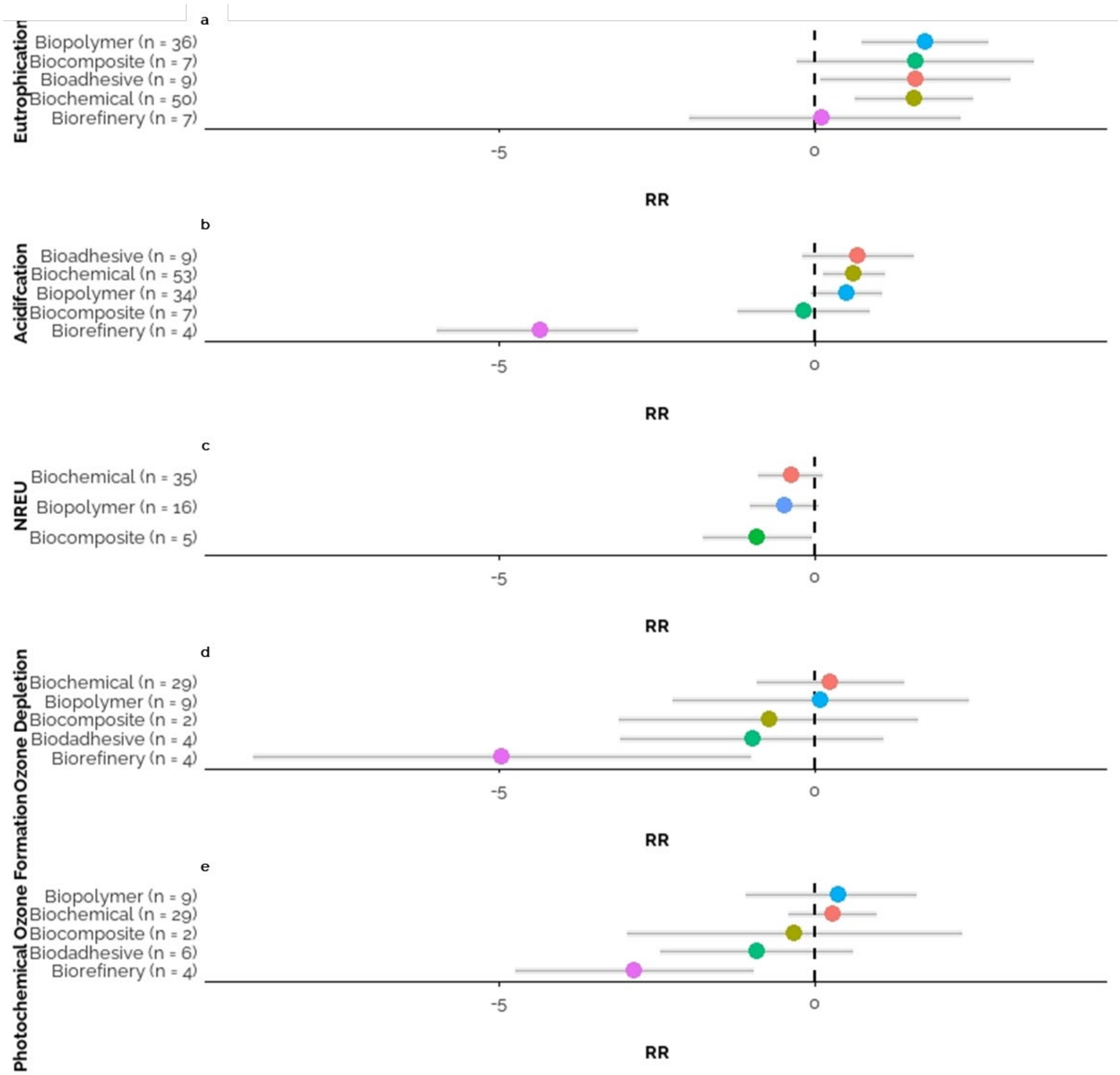


***Figure S.8****: LMM on relationship RR environmental footprints and product category: (a) Eutrophication: omnibus F: 0.46, p-value: 0.76; (b) Acidification: omnibus F: 9.08, p-value: 2.20E-05*; (c) NREU: omnibus F: 0.50, p-value: 0.61; (d) Ozone Depletion: omnibus F: 1.44, p-value: 0.26; (e) Photochemical Ozone Formation: omnibus F: 2.62, p-value: 0.047*.*

## S.9 Overview of arithmetic average RRs and corresponding 95% CI for greenhouse gas emissions, eutrophication, acidification, non-renewable energy-use, ozone depletion and photochemical ozone formation impacts

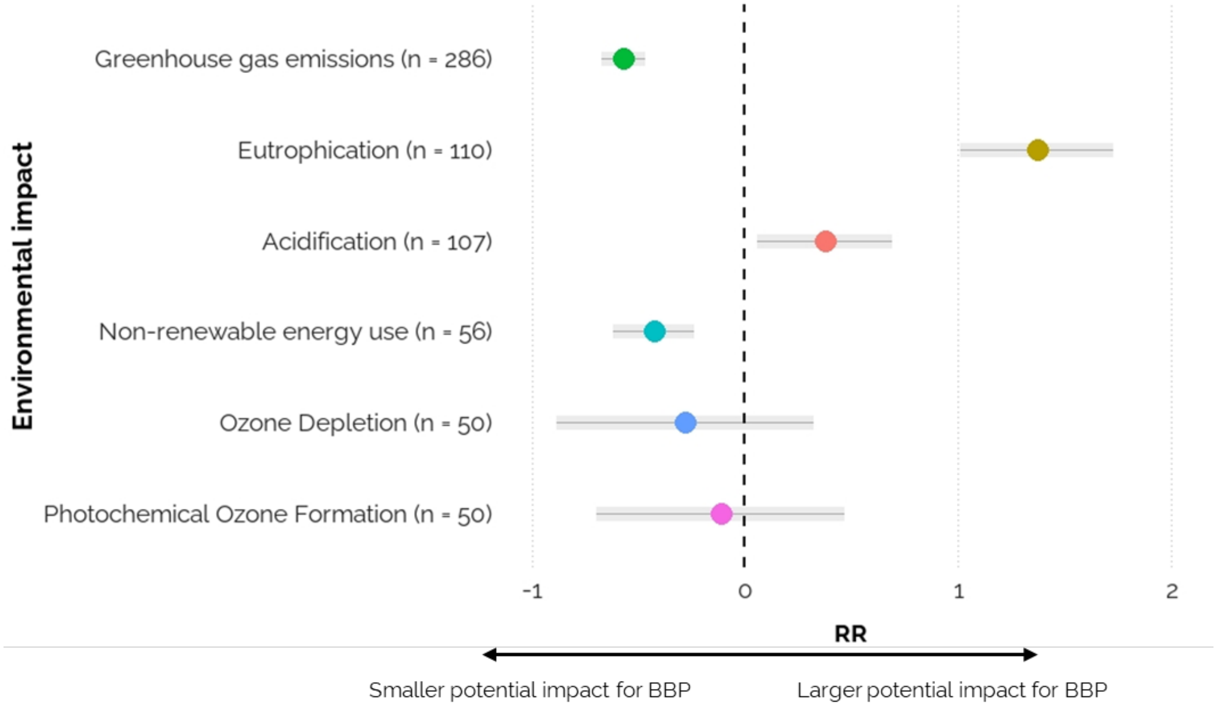


***Figure S.9****: Plot showing overall* ***arithmetic averages*** *and 95% CI of GHG, Eutrophication, Acidification, NREU, Ozone Depletion and Photochemical Ozone Formation impacts. Both arithmetic averages and predicted mean RRs from LMM (Figure 3 in the main text) show the same trends. In percentages, on average the GHG footprint is reduced with 43% (95% CI: 37, 49%), eutrophication is increased with 293% (95% CI: 175, 464%), acidification is increased with 45% (95% CI: 6, 99%), NREU is reduced with 35% (95% CI: 21, 46%), ozone depletion is reduced with 24% (95% CI: -59, 38%) and photochemical ozone formation is reduced with 11% (95% CI: -50, 60%).*

**S.10 Overview of the predicted mean and 95% CI of the RRs across all product types and studies for all environmental impacts with n ≤ 30. Separate random-effects models were ran for each impact category.**

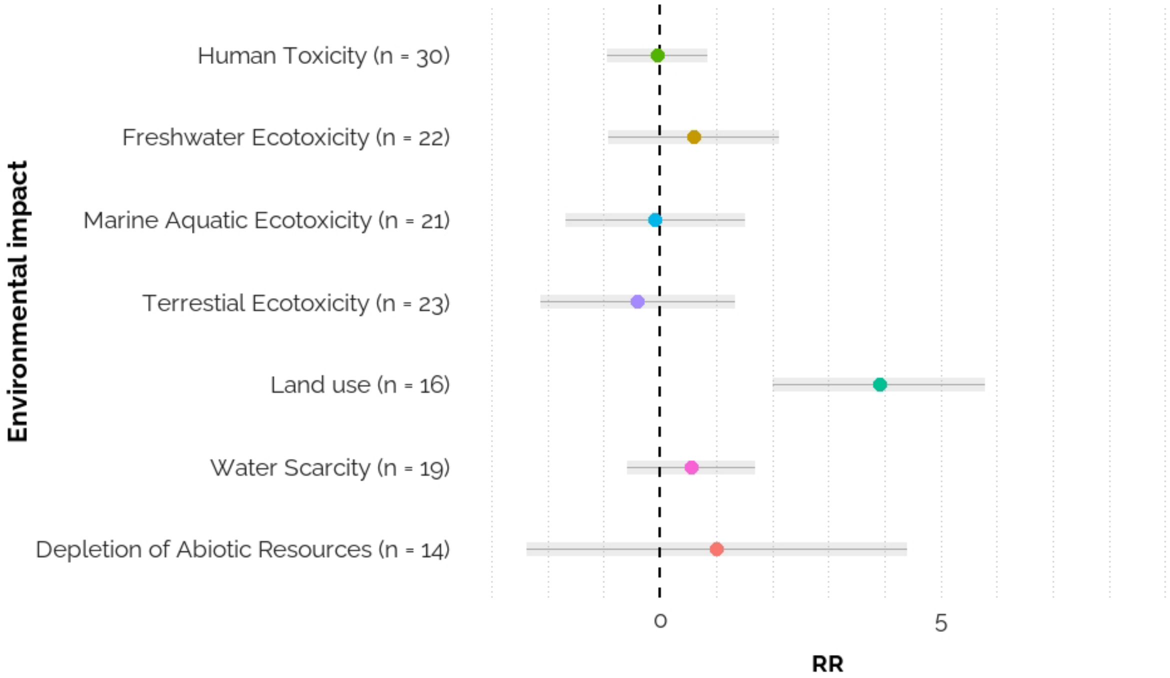


***Figure S.10****: plot showing predicted mean and 95% CI of the other environmental impacts' RRs (with n ≤ 30) collected from the 130 studies (Human Ecotoxicity, Freshwater Ecotoxicity, Marine Aquatic Ecotoxicity, Land Use, Water Scarcity, Depletion of Abiotic Resources).*

## References


1. Moni, S. M., Mahmud, R., High, K. & Carbajales-Dale, M. Life cycle assessment of emerging technologies: A review. *J. Ind. Ecol.* **24**, 52–63 (2020).

2. Ritchie, H. & Roser, M. $CO_2$ and Greenhouse Gas Emissions. *Our World in Data.* https://ourworldindata.org/co2-and-other-greenhouse-gas-emissions (2020).

3. Zheng, J. & Suh, S. Strategies to reduce the global carbon footprint of plastics. *Nat. Clim. Chang.* **9**, 374–378 (2019).

4. Galán-Martín, Á. *et al.* Sustainability footprints of a renewable carbon transition for the petrochemical sector within planetary boundaries. *One Earth* **4**, 565–583 (2021).

5. Levi, P. G. & Cullen, J. M. Mapping Global Flows of Chemicals: From Fossil Fuel Feedstocks to Chemical Products. *Environ. Sci. Technol.* **52**, (2018).

6. Ecoinvent. Ecoinvent database (Version 3.7). (2020).

## Database References


1. Urban, R. A. & Bakshi, B. R. 1,3-Propanediol from fossils versus biomass: A life cycle evaluation of emissions and ecological resources. Ind. Eng. Chem. Res. 48, 8068–8082 (2009).

2. Aryapratama, R. & Janssen, M. Prospective life cycle assessment of bio-based adipic acid production from forest residues. J. Clean. Prod. 164, 434–443 (2017).

3. Benalcázar, E. A., Deynoot, B. G., Noorman, H., Osseweijer, P. & Posada, J. A. Production of bulk chemicals from lignocellulosic biomass via thermochemical conversion and syngas fermentation: a comparative techno-economic and environmental assessment of different site-specific supply chain configurations. Biofuels, Bioprod. Biorefining 11, 861–886 (2017).

4. Ekman, A. & Börjesson, P. Environmental assessment of propionic acid produced in an agricultural biomass-based biorefinery system. J. Clean. Prod. 19, 1257–1265 (2011).

5. Liptow, C. & Tillman, A.-M. A Comparative Life Cycle Assessment Study of Polyethylene Based on Sugarcane and Crude Oil. J. Ind. Ecol. 16, 420–435 (2012).

6. Liptow, C., Tillman, A. M., Janssen, M., Wallberg, O. & Taylor, G. A. Ethylene based on woody biomass - What are environmental key issues of a possible future Swedish production on industrial scale. Int. J. Life Cycle Assess. 18, 1071–1081 (2013).

7. Tufvesson, P., Ekman, A., Sardari, R. R. R., Engdahl, K. & Tufvesson, L. Economic and environmental assessment of propionic acid production by fermentation using different renewable raw materials. Bioresour. Technol. 149, 556–564 (2013).

8. Zhang, Y., Hu, G. & Brown, R. C. Life cycle assessment of commodity chemical production from forest residue via fast pyrolysis. Int. J. Life Cycle Assess. 19, 1371–1381 (2014).

9. Fiorentino, G., Ripa, M., Mellino, S., Fahd, S. & Ulgiati, S. Life cycle assessment of Brassica carinata biomass conversion to bioenergy and platform chemicals. J. Clean. Prod. 66, 174–187 (2014).

10. Adom, F., Dunn, J. B., Han, J. & Sather, N. Life-cycle fossil energy consumption and greenhouse gas emissions of bioderived chemicals and their conventional counterparts. Environ. Sci. Technol. 48, 14624–14631 (2014).

11. Cok, B., Tsiropoulos, I., Roes, A. L. & Patel, M. K. Succinic acid production derived from carbohydrates: An energy and greenhouse gas assessment of a platform chemical toward a bio-based economy. Biofuels, Bioprod. Biorefining 8, 16–29 (2014).

12. Khoo, H. H., Wong, L. L., Tan, J., Isoni, V. & Sharratt, P. Synthesis of 2-methyl tetrahydrofuran from various lignocellulosic feedstocks: Sustainability assessment via LCA. Resour. Conserv. Recycl. 95, 174–182 (2015).

13. Gonzalez-Garay, A., Gonzalez-Miquel, M. & Guillen-Gosalbez, G. High-Value Propylene Glycol from Low-Value Biodiesel Glycerol: A Techno-Economic and Environmental Assessment under Uncertainty. ACS Publ. 5, 5723–5732 (2017).

14. Gunukula, S., Runge, T. & Anex, R. Assessment of Biocatalytic Production Parameters to Determine Economic and Environmental Viability. ACS Sustain. Chem. Eng. 5, 8119–8126 (2017).

15. Mandegari, M. A., Farzad, S., van Rensburg, E. & Görgens, J. F. Multi-criteria analysis of a biorefinery for co-production of lactic acid and ethanol from sugarcane lignocellulose. Biofuels, Bioprod. Biorefining 11, 971–990 (2017).

16. Zucaro, A., Forte, A. & Fierro, A. Greenhouse gas emissions and non-renewable energy use profiles of bio-based succinic acid from Arundo donax L. lignocellulosic feedstock. Clean Technol. Environ. Policy 19, 2129–2143 (2017).

17. Chen, W. S., Strik, D. P. B. T. B., Buisman, C. J. N. & Kroeze, C. Production of Caproic Acid from Mixed Organic Waste: An Environmental Life Cycle Perspective. Environ. Sci. Technol. 51, 7159–7168 (2017).

18. Zheng, J. L., Zhu, Y. H., Zhu, M. Q., Sun, G. T. & Sun, R. C. Life-cycle assessment and techno-economic analysis of the utilization of bio-oil components for the production of three chemicals. Green Chem. 20, 3287–3301 (2018).

19. Tripodi, A. et al. Acetonitrile from Bioethanol Ammoxidation: Process Design from the Grass-Roots and Life Cycle

Analysis. ACS Sustain. Chem. Eng. 6, 5441–5451 (2018).

20. Yang, M., Tian, X. & You, F. Manufacturing Ethylene from Wet Shale Gas and Biomass: Comparative Technoeconomic Analysis and Environmental Life Cycle Assessment. Ind. Eng. Chem. Res. 57, 5980–5998 (2018).

21. Alonso-Fariñas, B., Gallego-Schmid, A., Haro, P. & Azapagic, A. Environmental assessment of thermo-chemical processes for bio-ethylene production in comparison with bio-chemical and fossil-based ethylene. J. Clean. Prod. 202, 817–829 (2018).

22. Brunklaus, B., Rex, E., Carlsson, E. & Berlin, J. The future of Swedish food waste: An environmental assessment of existing and prospective valorization techniques. J. Clean. Prod. 202, 1–10 (2018).

23. González-García, S., Argiz, L., Míguez, P. & Gullón, B. Exploring the production of bio-succinic acid from apple pomace using an environmental approach. Chem. Eng. J. 350, 982–991 (2018).

24. Jonker, J. G. G. et al. Economic performance and GHG emission intensity of sugarcane- and eucalyptus-derived biofuels and biobased chemicals in Brazil. Biofuels, Bioprod. Biorefining 13, 950–977 (2019).

25. Kim, H., Choi, J., Park, J. & Won, W. Production of a sustainable and renewable biomass-derived monomer: conceptual process design and techno-economic analysis. Green Chem. 22, 7070–7079 (2020).

26. Wang, J., You, S., Lu, Z., Chen, R. & Xu, F. Life cycle assessment of bio-based levoglucosan production from cotton straw through fast pyrolysis. Bioresour. Technol. 307, 123179 (2020).

27. Lokesh, K. et al. Hybridised sustainability metrics for use in life cycle assessment of bio-based products: Resource efficiency and circularity. Green Chem. 22, 803–813 (2020).

28. van Duuren, J. B. J. H. et al. A limited LCA of bio-adipic acid: Manufacturing the nylon-6,6 precursor adipic acid using the benzoic acid degradation pathway from different feedstocks. Biotechnol. Bioeng. 108, 1298–1306 (2011).

29. Ögmundarson, Ó., Sukumara, S., Laurent, A. & Fantke, P. Environmental hotspots of lactic acid production systems. GCB Bioenergy 12, 19–38 (2020).

30. Yang, M. & Rosentrater, K. A. Life Cycle Assessment and Techno-Economic Analysis of Pressure Sensitive Bio-Adhesive Production. Energies 2019, Vol. 12, Page 4502 12, 4502 (2019).

31. Yang, M. & Rosentrater, K. A. Cradle-to-gate life cycle assessment of structural bio-adhesives derived from glycerol. Int. J. Life Cycle Assess. 26, 799–806 (2021).

32. Arias, A., González-García, S., González-Rodríguez, S., Feijoo, G. & Moreira, M. T. Cradle-to-gate Life Cycle Assessment of bio-adhesives for the wood panel industry. A comparison with petrochemical alternatives. Sci. Total Environ. 738, 140357 (2020).

33. Le Duigou, A., Deux, J. M., Davies, P. & Baley, C. PLLA/flax mat/balsa bio-sandwich-environmental impact and simplified life cycle analysis. Appl. Compos. Mater. 19, 363–378 (2012).

34. Mahalle, L., Alemdar, A., Mihai, M. & Legros, N. A cradle-to-gate life cycle assessment of wood fibre-reinforced polylactic acid (PLA) and polylactic acid/thermoplastic starch (PLA/TPS) biocomposites. Int. J. Life Cycle Assess. 19, 1305–1315 (2014).

35. Deng, Y. et al. Life cycle assessment of flax-fibre reinforced epoxidized linseed oil composite with a flame retardant for electronic applications. J. Clean. Prod. 133, 427–438 (2016).

36. Quintana, A., Alba, J., del Rey, R. & Guillén-Guillamón, I. Comparative Life Cycle Assessment of gypsum plasterboard and a new kind of bio-based epoxy composite containing different natural fibers. J. Clean. Prod. 185, 408–420 (2018).

37. Ita-Nagy, D., Vázquez-Rowe, I., Kahhat, R., Chinga-Carrasco, G. & Quispe, I. Reviewing environmental life cycle impacts of biobased polymers: current trends and methodological challenges. International Journal of Life Cycle Assessment vol. 25 2169–2189 (2020).

38. Akiyama, M., Tsuge, T. & Doi, Y. Environmental life cycle comparison of polyhydroxyalkanoates produced from renewable carbon resources by bacterial fermentation. Polym. Degrad. Stab. 80, 183–194 (2003).

39. Kim, S. & Dale, B. E. Environmental aspects of ethanol derived from no-tilled corn grain: nonrenewable energy

consumption and greenhouse gas emissions. Biomass and Bioenergy 28, 475–489 (2005).

40. Broeren, M. L. M. et al. Environmental assessment of bio-based chemicals in early-stage development: a review of methods and indicators. Biofuels, Bioprod. Biorefining 11, 701–718 (2017).

41. Piccinno, F., Hischier, R., Seeger, S. & Som, C. Predicting the environmental impact of a future nanocellulose production at industrial scale: Application of the life cycle assessment scale-up framework. J. Clean. Prod. 174, 283–295 (2018).

42. Tan, L. et al. Combining ex-ante LCA and EHS screening to assist green design: A case study of cellulose nanocrystal foam. J. Clean. Prod. 178, 494–506 (2018).

43. Ekman, A. & Börjesson, P. Life cycle assessment of mineral oil-based and vegetable oil-based hydraulic fluids including comparison of biocatalytic and conventional production methods. Int. J. Life Cycle Assess. 16, 297–305 (2011).

44. Brière, R. et al. Life cycle assessment of the production of surface-active alkyl polyglycosides from acid-assisted ball-milled wheat straw compared to the conventional production based on corn-starch. Green Chem. 20, 2135–2141 (2018).

45. Montazeri, M. & Eckelman, M. J. Life cycle assessment of UV-Curable bio-based wood flooring coatings. J. Clean. Prod. 192, 932–939 (2018).

46. Adom, F. K. & Dunn, J. B. Life cycle analysis of corn-stover-derived polymer-grade l-lactic acid and ethyl lactate: greenhouse gas emissions and fossil energy consumption. Biofuels, Bioprod. Biorefining 11, 258–268 (2017).

47. Garcia Gonzalez, M. N., Levi, M. & Turri, S. Development of polyester binders for the production of sustainable polyurethane coatings: Technological characterization and life cycle assessment. J. Clean. Prod. 164, 171–178 (2017).

48. Kikuchi, Y., Oshita, Y., Mayumi, K. & Hirao, M. Greenhouse gas emissions and socioeconomic effects of biomass-derived products based on structural path and life cycle analyses: A case study of polyethylene and polypropylene in Japan. J. Clean. Prod. 167, 289–305 (2017).

49. de Léis, C. M., Nogueira, A. R., Kulay, L. & Tadini, C. C. Environmental and energy analysis of biopolymer film based on cassava starch in Brazil. J. Clean. Prod. 143, 76–89 (2017).

50. Petrucci, R. et al. Life Cycle Analysis of Extruded Films Based on Poly(lactic acid)/Cellulose Nanocrystal/Limonene: A Comparative Study with ATBC Plasticized PLA/OMMT Systems. J. Polym. Environ. 26, 1891–1902 (2018).

51. Zhang, D., del Rio-Chanona, E. A., Wagner, J. L. & Shah, N. Life cycle assessments of bio-based sustainable polylimonene carbonate production processes. Sustain. Prod. Consum. 14, 152–160 (2018).

52. García González, M. N., Börjesson, P., Levi, M. & Turri, S. Development and Life Cycle Assessment of Polyester Binders Containing 2,5-Furandicarboxylic Acid and Their Polyurethane Coatings. J. Polym. Environ. 26, 3626–3637 (2018).

53. Cheroennet, N., Pongpinyopap, S., Leejarkpai, T. & Suwanmanee, U. A trade-off between carbon and water impacts in bio-based box production chains in Thailand: A case study of PS, PLAS, PLAS/starch, and PBS. J. Clean. Prod. 167, 987–1001 (2017).

54. Patel, M. K. et al. Second-generation bio-based plastics are becoming a reality - Non-renewable energy and greenhouse gas (GHG) balance of succinic acid-based plastic end products made from lignocellulosic biomass. Biofuels, Bioprod. Biorefining 12, 426–441 (2018).

55. Warlin, N. et al. A rigid spirocyclic diol from fructose-based 5-hydroxymethylfurfural: synthesis, life-cycle assessment, and polymerization for renewable polyesters and poly(urethane-urea)s. Green Chem. 21, 6667–6684 (2019).

56. Kookos, I. K., Koutinas, A. & Vlysidis, A. Life cycle assessment of bioprocessing schemes for poly(3-hydroxybutyrate) production using soybean oil and sucrose as carbon sources. Resour. Conserv. Recycl. 141, 317–328 (2019).

57. Manzardo, A. et al. Life Cycle Assessment Framework To Support the Design of Biobased Rigid Polyurethane Foams. ACS Omega 4, 14114–14123 (2019).

58. Mankar, S. V. et al. Synthesis, Life Cycle Assessment, and Polymerization of a Vanillin-Based Spirocyclic Diol toward Polyesters with Increased Glass-Transition Temperature. ACS Sustain. Chem. Eng. 7, 19090–19103 (2019).

59. Moretti, C., Junginger, M. & Shen, L. Environmental life cycle assessment of polypropylene made from used cooking oil.

Resour. Conserv. Recycl. 157, 104750 (2020).

60. Fridrihsone, A., Romagnoli, F., Kirsanovs, V. & Cabulis, U. Life Cycle Assessment of vegetable oil based polyols for polyurethane production. J. Clean. Prod. 266, 121403 (2020).

61. Parajuli, R. et al. Environmental impacts of producing bioethanol and biobased lactic acid from standalone and integrated biorefineries using a consequential and an attributional life cycle assessment approach. Sci. Total Environ. 598, 497–512 (2017).

62. Cai, H. et al. Life-cycle analysis of integrated biorefineries with co-production of biofuels and bio-based chemicals: co-product handling methods and implications. Biofuels, Bioprod. Biorefining 12, 815–833 (2018).

63. Fernandez-Dacosta, C. et al. Can we assess innovative bio-based chemicals in their early development stage? A comparison between early-stage and life cycle assessments. J. Clean. Prod. 230, 137–149 (2019).

64. Mercado, G., Dominguez, M., Herrera, I. & Melgoza, R. M. Are Polymers Toxic? Case Study: Environmental Impact of a Biopolymer. J. Environ. Sci. Eng. B 6, 121–126 (2017).

65. Vink, E. T. H. et al. The Sustainability of NatureWorksTM Polylactide Polymers and IngeoTM Polylactide Fibers: an Update of the Future. Macromol. Biosci. 4, 551–564 (2004).

66. Harding, K. G., Dennis, J. S., von Blottnitz, H. & Harrison, S. T. L. Environmental analysis of plastic production processes: Comparing petroleum-based polypropylene and polyethylene with biologically-based poly-β-hydroxybutyric acid using life cycle analysis. J. Biotechnol. 130, 57–66 (2007).

67. Pachón, E. R., Mandade, P. & Gnansounou, E. Conversion of vine shoots into bioethanol and chemicals: Prospective LCA of biorefinery concept. Bioresour. Technol. 303, 122946 (2020).

68. Vera, I., Hoefnagels, R., van der Kooij, A., Moretti, C. & Junginger, M. A carbon footprint assessment of multi-output biorefineries with international biomass supply: a case study for the Netherlands. Biofuels, Bioprod. Biorefining 14, 198–224 (2020).

69. Zah, R., Hischier, R., Leão, A. L. & Braun, I. Curauá fibers in the automobile industry – a sustainability assessment. J. Clean. Prod. 15, 1032–1040 (2007).

70. Kim, S., Dale, B. E., Drzal, L. T. & Misra, M. Life Cycle Assessment of Kenaf Fiber Reinforced Biocomposite. J. Biobased Mater. Bioenergy 2, 85–93 (2008).

71. De Figueirêdo, M. C. B. et al. Life cycle assessment of cellulose nanowhiskers. J. Clean. Prod. 35, 130–139 (2012).

72. Li, Q., McGinnis, S., Sydnor, C., Wong, A. & Renneckar, S. Nanocellulose life cycle assessment. ACS Sustain. Chem. Eng. 1, 919–928 (2013).

73. Manda, B. M. K., Worrell, E. & Patel, M. K. Prospective life cycle assessment of an antibacterial T-shirt and supporting business decisions to create value. Resour. Conserv. Recycl. 103, 47–57 (2015).

74. Piccinno, F., Hischier, R., Seeger, S. & Som, C. Life cycle assessment of a new technology to extract, functionalize and orient cellulose nanofibers from food waste. ACS Sustain. Chem. Eng. 3, 1047–1055 (2015).

75. Hervy, M., Evangelisti, S., Lettieri, P. & Lee, K. Y. Life cycle assessment of nanocellulose-reinforced advanced fibre composites. Compos. Sci. Technol. 118, 154–162 (2015).

76. Tufvesson, L. M. & Börjesson, P. Wax production from renewable feedstock using biocatalysts instead of fossil feedstock and conventional methods. Int. J. Life Cycle Assess. 13, 328–338 (2008).

77. Bernier, E., Lavigne, C. & Robidoux, P. Y. Life cycle assessment of kraft lignin for polymer applications. Int. J. Life Cycle Assess. 18, 520–528 (2013).

78. Deng, Y., Achten, W. M. J., Van Acker, K. & Duflou, J. R. Life cycle assessment of wheat gluten powder and derived packaging film. Biofuels, Bioprod. Biorefining 7, 429–458 (2013).

79. Pang, M. M., Pun, M. Y., Chow, W. S. & Ishak, Z. A. M. Carbon footprint calculation for thermoformed starch-filled polypropylene biobased materials. J. Clean. Prod. 64, 602–608 (2014).

80. Frascari, D., Molina Bacca, A. E., Wardenaar, T., Oertlé, E. & Pinelli, D. Continuous flow adsorption of phenolic compounds from olive mill wastewater with resin XAD16N: life cycle assessment, cost–benefit analysis and process optimization. J. Chem. Technol. Biotechnol. 94, 1968–1981 (2019).

81. Ghasemi, S., Sibi, M. P., Ulven, C. A., Webster, D. C. & Pourhashem, G. A Preliminary Environmental Assessment of Epoxidized Sucrose Soyate (ESS)-Based Biocomposite. Mol. 2020, Vol. 25, Page 2797 25, 2797 (2020).

82. Sadhukhan, J. et al. Novel macroalgae (seaweed) biorefinery systems for integrated chemical, protein, salt, nutrient and mineral extractions and environmental protection by green synthesis and life cycle sustainability assessments. Green Chem. 21, 2635–2655 (2019).

83. Lin, Z., Nikolakis, V. & Ierapetritou, M. Life cycle assessment of biobased p -xylene production. Ind. Eng. Chem. Res. 54, 2366–2378 (2015).

84. Dros, A. B., Larue, O., Reimond, A., De Campo, F. & Pera-Titus, M. Hexamethylenediamine (HMDA) from fossil- vs. bio-based routes: an economic and life cycle assessment comparative study. Green Chem. 17, 4760–4772 (2015).

85. Liptow, C., Tillman, A. M. & Janssen, M. Life cycle assessment of biomass-based ethylene production in Sweden — is gasification or fermentation the environmentally preferable route? Int. J. Life Cycle Assess. 20, 632–644 (2015).

86. Khoo, H. H., Ee, W. L. & Isoni, V. Bio-chemicals from lignocellulose feedstock: Sustainability, LCA and the green conundrum. Green Chem. 18, 1912–1922 (2016).

87. Forte, A., Zucaro, A., Basosi, R. & Fierro, A. LCA of 1,4-Butanediol Produced via Direct Fermentation of Sugars from Wheat Straw Feedstock within a Territorial Biorefinery. Mater. 2016, Vol. 9, Page 563 9, 563 (2016).

88. Montazeri, M. & Eckelman, M. J. Life Cycle Assessment of Catechols from Lignin Depolymerization. ACS Sustain. Chem. Eng. 4, 708–718 (2016).

89. Gezae Daful, A. & Görgens, J. F. Techno-economic analysis and environmental impact assessment of lignocellulosic lactic acid production. Chem. Eng. Sci. 162, 53–65 (2017).

90. Moussa, H. I., Elkamel, A. & Young, S. B. Assessing energy performance of bio-based succinic acid production using LCA. J. Clean. Prod. 139, 761–769 (2016).

91. Morales, M. et al. Sustainability assessment of succinic acid production technologies from biomass using metabolic engineering. Energy Environ. Sci. 9, 2794–2805 (2016).

92. Cespi, D., Passarini, F., Vassura, I. & Cavani, F. Butadiene from biomass, a life cycle perspective to address sustainability in the chemical industry. Green Chem. 18, 1625–1638 (2016).

93. Gargalo, C. L. et al. Assessing the environmental sustainability of early stage design for bioprocesses under uncertainties: An analysis of glycerol bioconversion. J. Clean. Prod. 139, 1245–1260 (2016).

94. Morales, M. et al. Environmental and economic assessment of lactic acid production from glycerol using cascade bio- and chemocatalysis. Energy Environ. Sci. 8, 558–567 (2015).

95. Yu, J. & Chen, L. X. L. The Greenhouse Gas Emissions and Fossil Energy Requirement of Bioplastics from Cradle to Gate of a Biomass Refinery. Environ. Sci. Technol. 42, 6961–6966 (2008).

96. Kim, S. & Dale, B. E. Energy and greenhouse gas profiles of polyhydroxybutyrates derived from corn grain: A life cycle perspective. Environ. Sci. Technol. 42, 7690–7695 (2008).

97. Kendall, A. A life cycle assessment of biopolymer production from material recovery facility residuals. Resour. Conserv. Recycl. 61, 69–74 (2012).

98. Eerhart, A. J. J. E., Faaij, A. P. C. & Patel, M. K. Replacing fossil based PET with biobased PEF; Process analysis, energy and GHG balance. Energy Environ. Sci. 5, 6407–6422 (2012).

99. Nuss, P. & Gardner, K. H. Attributional life cycle assessment (ALCA) of polyitaconic acid production from northeast US softwood biomass. Int. J. Life Cycle Assess. 18, 603–612 (2013).

100. Akanuma, Y., Selke, S. E. M. & Auras, R. A preliminary LCA case study: Comparison of different pathways to produce purified terephthalic acid suitable for synthesis of 100 % bio-based PET. Int. J. Life Cycle Assess. 19, 1238–1246 (2014).

101. Papong, S. et al. Comparative assessment of the environmental profile of PLA and PET drinking water bottles from a life cycle perspective. J. Clean. Prod. 65, 539–550 (2014).

102. Hohenschuh, W., Kumar, D. & Murthy, G. S. Economic and cradle-to-gate life cycle assessment of poly-3-hydroxybutyrate production from plastic producing, genetically modified hybrid poplar leaves. J. Renew. Sustain. Energy 6, 063113 (2014).

103. Heimersson, S., Morgan-Sagastume, F., Peters, G. M., Werker, A. & Svanström, M. Methodological issues in life cycle assessment of mixed-culture polyhydroxyalkanoate production utilising waste as feedstock. N. Biotechnol. 31, 383–393 (2014).

104. Zolkarnain, N. et al. Evaluation of environmental impacts and GHG of palm polyol production using life cycle assessment approach. J. oil Palm Res. 27, 144–155 (2015).

105. Sun, X. Z., Minowa, T., Yamaguchi, K. & Genchi, Y. Evaluation of energy consumption and greenhouse gas emissions from poly(phenyllactic acid) production using sweet sorghum. J. Clean. Prod. 87, 208–215 (2015).

106. Tecchio, P., Freni, P., De Benedetti, B. & Fenouillot, F. Ex-ante Life Cycle Assessment approach developed for a case study on bio-based polybutylene succinate. J. Clean. Prod. 112, 316–325 (2016).

107. Gontia, P. & Janssen, M. Life cycle assessment of bio-based sodium polyacrylate production from pulp mill side streams: case study of thermo-mechanical and sulfite pulp mills. J. Clean. Prod. 131, 475–484 (2016).

108. Chen, L., Pelton, R. E. O. & Smith, T. M. Comparative life cycle assessment of fossil and bio-based polyethylene terephthalate (PET) bottles. J. Clean. Prod. 137, 667–676 (2016).

109. Posen, I. D., Jaramillo, P. & Griffin, W. M. Uncertainty in the Life Cycle Greenhouse Gas Emissions from U.S. Production of Three Biobased Polymer Families. Environ. Sci. Technol. 50, 2846–2858 (2016).

110. Belboom, S. & Léonard, A. Does biobased polymer achieve better environmental impacts than fossil polymer? Comparison of fossil HDPE and biobased HDPE produced from sugar beet and wheat. Biomass and Bioenergy 85, 159–167 (2016).

111. Kim, S. & Dale, B. E. Life Cycle Assessment Study of Biopolymers (Polyhydroxyalkanoates) - Derived from No-Tilled Corn (11 pp). Int. J. Life Cycle Assess. 2005 103 10, 200–210 (2004).

112. Ingrao, C. et al. Spent-coffee grounds as a zero-burden material blended with bio-based poly(butylene succinate) for production of bio-composites: Findings from a Life Cycle Assessment application experience. Environ. Impact Assess. Rev. 97, 106919 (2022).

113. Zuiderveen, E. A. R., Ansovini, D., Gruter, G.-J. M. & Shen, L. Ex-ante life cycle assessment of polyethylenefuranoate (PEF) from bio-based monomers synthesized via a novel electrochemical process. Clean. Environ. Syst. 2, 100036 (2021).

114. Haus, M. O., Winter, B., Fleitmann, L., Palkovits, R. & Bardow, A. Making more from bio-based platforms: life cycle assessment and techno-economic analysis of N-vinyl-2-pyrrolidone from succinic acid. Green Chem. 24, 6671–6684 (2022).

115. Quinteiro, P., Gama, N. V., Ferreira, A., Dias, A. C. & Barros-Timmons, A. Environmental assessment of different strategies to produce rigid polyurethane foams using unrefined crude glycerol. J. Clean. Prod. 371, 133554 (2022).

116. Yang, F., Meerman, H., Zhang, Z., Jiang, J. & Faaij, A. Integral techno-economic comparison and greenhouse gas balances of different production routes of aromatics from biomass with CO2 capture. J. Clean. Prod. 372, 133727 (2022).

117. Fazeni-Fraisl Energieinstitut, K. et al. Comparative life cycle assessment of first- and second-generation bio-isobutene as a drop-in substitute for fossil isobutene. Biofuels, Bioprod. Biorefining 17, 207–225 (2023).

118. Boekaerts, B. et al. Assessment of the environmental sustainability of solvent-less fatty acid ketonization to bio-based ketones for wax emulsion applications. Green Chem. 23, 7137–7161 (2021).

119. Müller-Carneiro, J., Figueirêdo, M. C. B. de, Rodrigues, C., Azeredo, H. M. C. de & Freire, F. Ex-ante life cycle assessment framework and application to a nano-reinforced biopolymer film based on mango kernel. Resour. Conserv. Recycl. 188,

106637 (2023).

120. Gian, M., García-Velásquez, C. & van der Meer, Y. Comparative life cycle assessment of the biochemical and thermochemical production routes of biobased terephthalic acid using Miscanthus in the Netherlands. Clean. Environ. Syst. 6, 100085 (2022).

121. Guiton, M. et al. Comparative Life Cycle Assessment of a microalgae-based oil metal working fluid with its petroleum-based and vegetable-based counterparts. J. Clean. Prod. 338, 130506 (2022).

122. Gadkari, S., Kumar, D., Qin, Z. hao, Ki Lin, C. S. & Kumar, V. Life cycle analysis of fermentative production of succinic acid from bread waste. Waste Manag. 126, 861–871 (2021).

123. Rajendran, N. & Han, J. Techno-economic analysis and life cycle assessment of poly (butylene succinate) production using food waste. Waste Manag. 156, 168–176 (2023).

124. García-Velásquez, C. & van der Meer, Y. Mind the Pulp: Environmental and economic assessment of a sugar beet pulp biorefinery for biobased chemical production. Waste Manag. 155, 199–210 (2023).

125. Kim, T., Bamford, J., Gracida-Alvarez, U. R. & Benavides, P. T. Life Cycle Greenhouse Gas Emissions and Water and Fossil-Fuel Consumptions for Polyethylene Furanoate and Its Coproducts from Wheat Straw. ACS Sustain. Chem. Eng. 10, 2830–2843 (2022).

126. Kim, T., Bhatt, A., Tao, L. & Benavides, P. T. Life cycle analysis of polylactic acids from different wet waste feedstocks. J. Clean. Prod. 380, 135110 (2022).

127. Saavedra del Oso, M., Mauricio-Iglesias, M., Hospido, A. & Steubing, B. Prospective LCA to provide environmental guidance for developing waste-to-PHA biorefineries. J. Clean. Prod. 383, 135331 (2023).

128. Ayala, M., Thomsen, M. & Pizzol, M. Life Cycle Assessment of pilot scale production of seaweed-based bioplastic. Algal Res. 71, 103036 (2023).

129. Carcassi, O. B. et al. Carbon Footprint Assessment of a Novel Bio-Based Composite for Building Insulation. Sustain. 2022, Vol. 14, Page 1384 14, 1384 (2022).

130. Alvarenga, R. A. et al. Life cycle assessment of bioethanol-based PVC. Biofuels, Bioprod. Biorefining 7, 386–395 (2013).
